# A186 ASSOCIATION OF ULCERATIVE COLITIS BOWEL URGENCY IMPROVEMENT WITH CLINICAL RESPONSE AND REMISSION

**DOI:** 10.1093/jcag/gwac036.186

**Published:** 2023-03-07

**Authors:** D B Clemow, C Sapin, T Hibi, M C Dubinsky, S Vermeire, S Schreiber, T H Gibble, L Peyrin-Biroulet, M Watanabe, R Panaccione, J Jones

**Affiliations:** 1 Eli Lilly and Company, Indianapolis, United States; 2 Kitasato Institute, Keio University School of Medicine, Tokyo, Japan; 3 Mount Sinai Hospital, New York, United States; 4 University Hospitals Leuven, Leuven, Belgium; 5 University Hospital Schleswig-Holstein, Kiel, Germany; 6 Nancy University Hospital, Vandœuvre-lès-Nancy, France; 7 Tokyo Medical and Dental University, Tokyo, Japan; 8 University of Calgary, Calgary; 9 Division of Digestive Care and Endoscopy, Department of Medicine, Department of Community Health and Epidemiology, Dalhousie University, Halifax, Canada

## Abstract

**Background:**

Ulcerative colitis (UC) can result in a high prevalence of bowel movement urgency (BU), significantly reducing patient quality of life.

**Purpose:**

Early BU improvement association with later clinical endpoint improvements was examined in moderately-to-severely active UC patients (pts) treated with mirikizumab (miri).

**Method:**

BU was evaluated in Phase 3 randomized placebo (PBO)-controlled 12-week induction (LUCENT-1, NCT03518086) and 40-week maintenance (LUCENT-2, NCT03524092) trials with miri. Pts received IV miri 300mg or PBO during induction. Week (W)12 miri responders were rerandomized at LUCENT-2 baseline (BL) to subcutaneous miri 200mg or PBO. BU was measured with 11-point Urgency Numeric Rating Scale (UNRS) from 0 (no urgency) to 10 (worst possible). Pts’ UNRS scores were an average from 7 consecutive days prior to visit. Association of pts with BU Clinically Meaningful Improvement (CMI) or BU remission between BL and W4 with the proportion of pts achieving clinical response, and clinical, endoscopic, or symptomatic remission at end of W12 was assessed. For pts who achieved clinical response at W12, the analyses were repeated for the end of maintenance based on W12 BU status. Logistic regression models with treatment, urgency (BU CMI or BU Remission), treatment-by-urgency group interaction, and stratification factors were fitted to examine the association between early urgency improvement and later clinical endpoints.

**Result(s):**

Treatment-by-urgency group interactions were not statistically significant across clinical outcomes for induction and maintenance. For induction, treatment and urgency status were statistically significant. Pts experiencing BU CMI or BU remission at W4 were consistently more likely to achieve clinical response, and clinical, endoscopic, or symptomatic remission at W12 for both treatment groups. For remission, only treatment main effect was statistically significant. Among miri induction clinical responders (an enriched population), BU CMI or BU Remission at end of induction (W12) was not associated with later maintenance efficacy outcomes (W52). Miri-treated pts achieved higher rates of clinical response, and clinical, endoscopic, or symptomatic remission at W52 than with PBO regardless of BU CMI or BU Remission at W12 (Table).

**Image:**

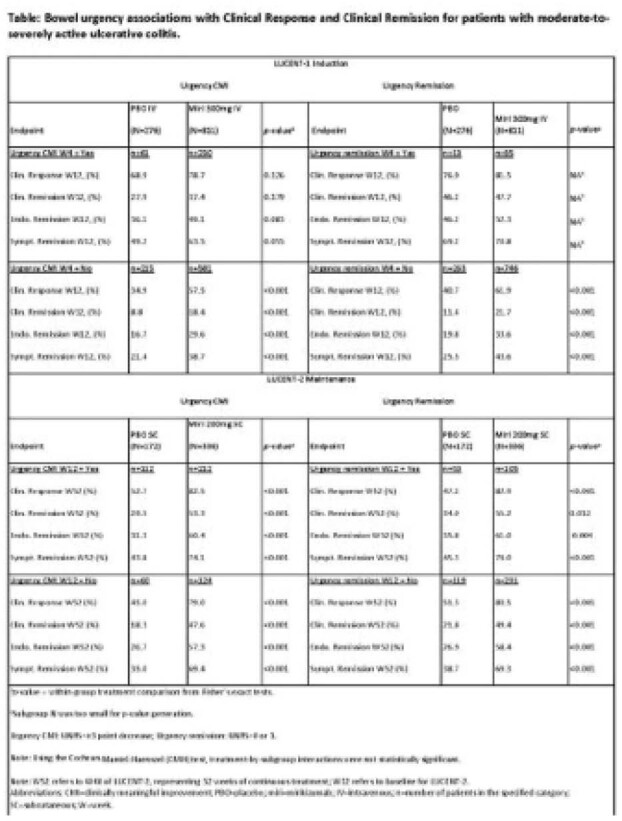

**Conclusion(s):**

Early BU Improvement, CMI or Remission, was associated with better clinical outcomes during induction for miri and PBO pts, showing BU is a sensitive predictor of early clinical outcomes. Among miri induction responders, miri consistently provided better maintenance of response and remission rates than PBO.

**Please acknowledge all funding agencies by checking the applicable boxes below:**

Other

**Please indicate your source of funding;:**

Eli Lilly and Company

**Disclosure of Interest:**

D. Clemow Employee of: Eli Lilly and Company, C. Sapin Employee of: Eli Lilly and Company, T. Hibi Grant / Research support from: AbbVie, ActivAid, Alfresa Pharma, Bristol Myers Squibb, Eli Lilly Japan K.K., Ferring Pharmaceuticals, Gilead Sciences, Janssen Pharmaceutical K.K., JMDC, Mochida Pharmaceutical, Nippon Kayaku, Pfizer Japan, and Takeda, Consultant of: AbbVie, Apo Plus Station, Bristol Myers Squibb, Celltrion, EA Pharma, Eli Lilly and Company, Gilead Sciences, Janssen, Kyorin, Mitsubishi Tanabe Pharma, Nichi-Iko Pharmaceutical, Pfizer, Takeda, and Zeria Pharmaceutical, Speakers bureau of: AbbVie, Aspen Japan K.K., Ferring Pharmaceuticals, Gilead Sciences, Janssen, JIMRO, Mitsubishi Tanabe Pharma, Mochida Pharmaceutical, Pfizer, and Takeda, M. Dubinsky Shareholder of: Trellus Health, Grant / Research support from: AbbVie, Janssen, Pfizer, and Prometheus Biosciences, Consultant of: AbbVie, Arena Pharmaceuticals, Boehringer Ingelheim, Bristol Myers Squibb, Celgene, Eli Lilly and Company, F. Hoffmann-La Roche, Genentech, Gilead Sciences, Janssen, Pfizer, Prometheus Therapeutics and Diagnostics, Takeda, and UCB Pharma, S. Vermeire Consultant of: AbbVie, Arena Pharmaceuticals, Avaxia Biologics, Boehringer Ingelheim, Celgene, Dr. Falk Pharma, Ferring Pharmaceuticals, Galapagos NV, Genentech/Roche, Gilead Sciences, Hospira, Janssen, Mundipharma, Merck Sharp & Dohme, Pfizer, ProDigest, Progenity, Prometheus Therapeutics and Diagnostics, Robarts Clinical Trials, Second Genome, Shire, Takeda, Theravance Biopharma, and Tillots Pharma AG, Speakers bureau of: AbbVie, Dr. Falk Pharma, Ferring Pharmaceuticals, Galapagos NV, Genentech/Roche, Gilead Sciences, Janssen, Pfizer, Robarts Clinical Trials, and Takeda, S. Schreiber Grant / Research support from: personal fees and/or travel support from: AbbVie, Amgen, Arena Pharmaceuticals, Biogen, Bristol Myers Squibb, Celgene, Celltrion, Eli Lilly and Company, Dr. Falk Pharma, Ferring Pharmaceuticals, Fresenius Kabi, Galapagos NV, Gilead Sciences, I-MAB Biopharma, Janssen, Merck Sharp & Dohme, Mylan, Novartis, Pfizer, Protagonist Therapeutics, Provention Bio, Roche, Sandoz/Hexal, Shire, Takeda, Theravance Biopharma, and UCB Pharma, T. Gibble Employee of: Eli Lilly and Company, L. Peyrin-Biroulet Grant / Research support from: AbbVie, Fresenius Kabi, Merck Sharp & Dohme, and Takeda, Consultant of: AbbVie, Alimentiv, Allergan, Amgen, Arena Pharmaceuticals, Biogen, Bristol Myers Squibb, Celgene, Celltrion, Eli Lilly and Company, Enthera, Ferring Pharmaceuticals, Fresenius Kabi, Galapagos NV, Genentech, Gilead Sciences, Gossamer Bio, InDex Pharmaceuticals, Inotrem, Janssen, Merck Sharp & Dohme, Mylan, Norgine, Ono Pharmaceutical, OSE Immunotherapeutics, Pandion Therapeutics, Pfizer, Roche, Samsung Bioepis, Sandoz, Takeda, Theravance Biopharma, Thermo Fisher Scientific, Tillots Pharma AG, Viatris, and Vifor Pharma, M. Watanabe Grant / Research support from: AbbVie, Alfresa Pharma, EA Pharma, Kissei, Kyorin, Mitsubishi Tanabe Pharma, Mochida Pharmaceutical, Nippon Kayaku, Takeda, and Zeria Pharmaceutical, Consultant of: AbbVie, Boehringer Ingelheim, EA Pharma, Eli Lilly Japan K.K., Gilead Sciences, Nippon, and Takeda, Speakers bureau of: EA Pharma, Eli Lilly Japan K.K., Gilead Sciences, Janssen, JIMRO, Kissei, Mitsubishi Tanabe Pharma, Mochida Pharmaceutical, Pfizer Japan, Takeda, and Zeria Pharmaceutical, R. Panaccione Grant / Research support from: AbbVie, Ferring Pharmaceuticals, Janssen, Pfizer, and Takeda, Consultant of: Abbott, AbbVie, Alimentiv, Amgen, Arena Pharmaceuticals, AstraZeneca, Biogen, Boehringer Ingelheim, Bristol Myers Squibb, Celgene, Celltrion, Cosmo Pharmaceuticals, Eisai, Elan Pharma, Eli Lilly and Company, Ferring Pharmaceuticals, Galapagos NV, Genentech, Gilead Sciences, GlaxoSmithKline, Janssen, Merck, Mylan, Oppilan Pharma, Pandion Therapeutics, Pfizer, Progenity, Protagonist Therapeutics, Roche, Sandoz, Satisfai Health, Shire, Sublimity Therapeutics, Takeda, Theravance Biopharma, and UCB Pharma, J. Jones: None Declared

